# The effects of a dynamic patellar realignment brace on disease determinants for patellofemoral instability in the upright weight-bearing condition

**DOI:** 10.1186/s13018-015-0265-x

**Published:** 2015-08-19

**Authors:** Christoph Becher, Thees Schumacher, Benjamin Fleischer, Max Ettinger, Tomas Smith, Sven Ostermeier

**Affiliations:** Department of Orthopedic Surgery, Hannover Medical School, 1-7 Anna-von-Borries-Strasse, 30625 Hannover, Germany; Department of Biomechanics and Biomaterials, Hannover Medical School, Hannover, Germany; Gelenk Klinik, Gundelfingen, Freiburg Germany

**Keywords:** Knee, Patellar instability, MRI, Patellofemoral indices, Weight bearing, Realignment brace

## Abstract

**Background:**

Patellar stabilizing braces are used to alleviate pain and prevent subluxation/dislocation by having biomechanical effects in terms of improved patellar tracking. The purpose of this study is to analyze the effects of the dynamic patellar realignment brace, Patella Pro (Otto Bock GmbH, Duderstadt, Germany), on disease determinants in subjects with patellofemoral instability using upright weight-bearing magnetic resonance imaging (MRI).

**Methods:**

Twenty subjects (8 males and 12 females) with lateral patellofemoral instability were studied in an open-configuration magnetic resonance imaging scanner in an upright weight-bearing position at full extension (0° flexion) and 15° and 30° flexion with and without the realignment brace. Disease determinants were defined by common patellofemoral indices that included the Insall–Salvati Index, Caton–Deschamps Index, and the Patellotrochlear Index to determine patella height and patella tilt angle, bisect offset, and tuberositas tibiae–trochlear groove (TT–TG) distance to determine patellar rotation and translation with respect to the femur and the alignment of the extensor mechanism.

**Results:**

Analyses of variance revealed a significant effect of the brace with reduction of the three patellar height ratios, patella tilt angle, and bisect offset as well as TT–TG distance. Post hoc pairwise comparisons of the corresponding conditions with and without the realignment brace revealed significantly reduced patella height ratios, patella tilt angles, and bisect offsets at full extension and 15° and 30° flexion. No significant differences between the TT–TG distances at full extension but significant reductions at 15° and 30° flexion were observed when using the realignment brace compared to no brace.

**Conclusions:**

This study suggests that the dynamic patellar realignment brace is capable of improving disease determinants in the upright weight-bearing condition in the range of 0° to 30° flexion in patients with patellofemoral instability.

## Background

The etiology of lateral patellar instability is multifactorial with various contributing factors such as trochlear dysplasia, patella alta, malrotation as well as medial soft tissue disruption and insufficiency [1, 2]. Various parameters to define and quantify these factors have been described [3]. Radiological assessments with radiography, computed tomography (CT), and magnetic resonance imaging (MRI) are of high importance for further evaluation of these parameters to assist clinical decision-making and differential diagnosis [2–5].

MRI has evolved as the most essential method offering numerous options such as the examination during weight-bearing conditions and at different flexion angles [6–8]. This appears important since at extension and early flexion, patellar motion is largely influenced by quadriceps activation [6, 8–10] and most patients are prone to lateral subluxation and/or dislocations within the first 30° of flexion when the patella is not constrained by the trochlea [11].

Various conservative and operative options are available for the treatment of patellar instability. In conservative management, physical therapy represents an important factor since muscular dysfunction might be positively influenced [12–14]. Supportive devices such as patellar stabilizing braces and patellar taping are used to alleviate pain by restoring better joint kinematics and prevent subluxation/dislocation by having biomechanical effects in terms of improved patellar tracking [15–18] and improved proprioception [14, 19]. A new dynamic patellar realignment brace (Patella Pro, Otto Bock GmbH, Duderstadt, Germany) with a dynamic tracking system to apply a medially directed force on the patella was recently introduced and is currently being subjected to a clinical trial [20]. A biomedical study using human cadaver specimens showed that the brace has the potential to medialize the patella during 0°–45° flexion [21]. However, the biomechanical effects of the brace in vivo remain to be proven.

In this study, it was hypothesized that the dynamic patellar realignment brace Patella Pro has positive effects on typical disease determinants in subjects with lateral patellar instability defined by common patellofemoral indices used in MR imaging at 0°, 15°, and 30° flexion in the upright weight-bearing condition.

## Materials and methods

### Study design and study group

In this explorative case–control study, the participants had to fulfill the following inclusion criteria: age >16 years, a minimum of two lateral patellar dislocations, and demonstration of appropriate fit of the realignment brace with the patients’ anatomy. Exclusion criteria were inability to keep the target knee positions for the estimated examination time as evaluated by pre-testing, significant frontal plane femorotibial malalignment >7° as measured on long leg standing radiography in the clinical routine, pregnancy, body mass index >35 kg/m^2^, and implanted devices that could interact with the magnetic field of the MRI scanner. Accordingly, 20 subjects (8 males and 12 females) with lateral patellofemoral instability scheduled for realignment surgery were included. The average age was 25.3 ± 7.0 (17–39) years; the average BMI was 23.6 ± 3.7 (18–34) kg/m^2^. The gender distribution of the study sample (8 males and 12 females) was chosen disproportionally based on epidemiological data that females are more frequently affected by chronic patellofemoral instability [22]. Institutional review board approval (ID *blinded*) was obtained from the ethics committee of Hannover Medical School prior to the study.

### Patellar realignment brace (Patella Pro)

**Fig. 1:**
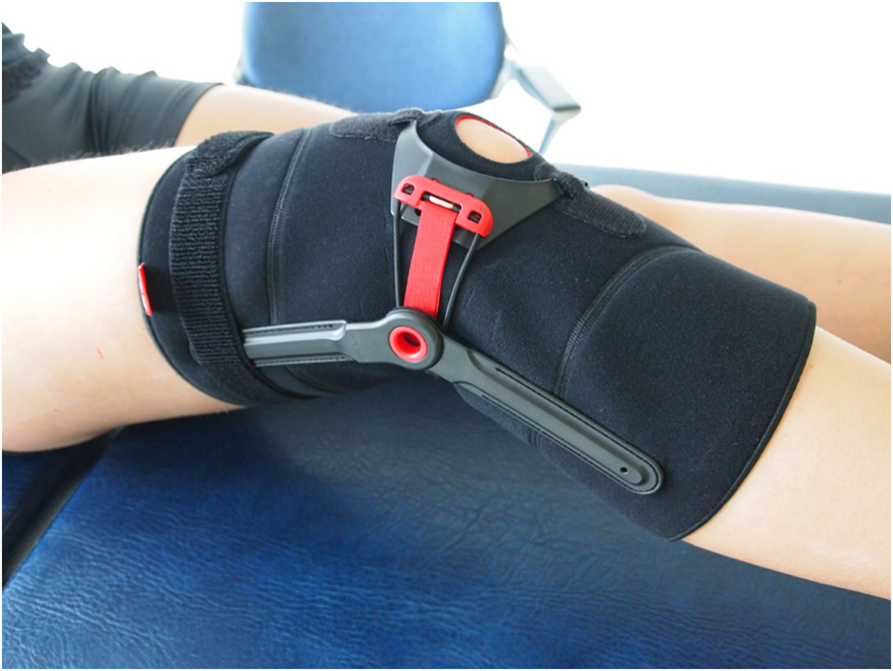
Patellar realignment brace (Patella Pro)

The patella brace used in this study was the Patella Pro dynamic realignment brace (Patella Pro, Otto Bock GmbH, Duderstadt, Germany). The brace is made of lightweight, breathable material with a unique vector grip that prevents the brace from slipping. It is equipped with a tracking system mounted on a hinged sleeve that can apply a dynamic, medially directed force on the patella within its range of motion (Fig. 1). The pressure exerted by the tracking system remains constant across the range of motion of the knee and does not increase with the flexion angle. The brace is available in five different sizes (XS, S, M, L, and XL) and can be fitted individually with hook and loop material and ratchet closures to ensure optimal function.

### MRI assessment

**Fig. 2:**
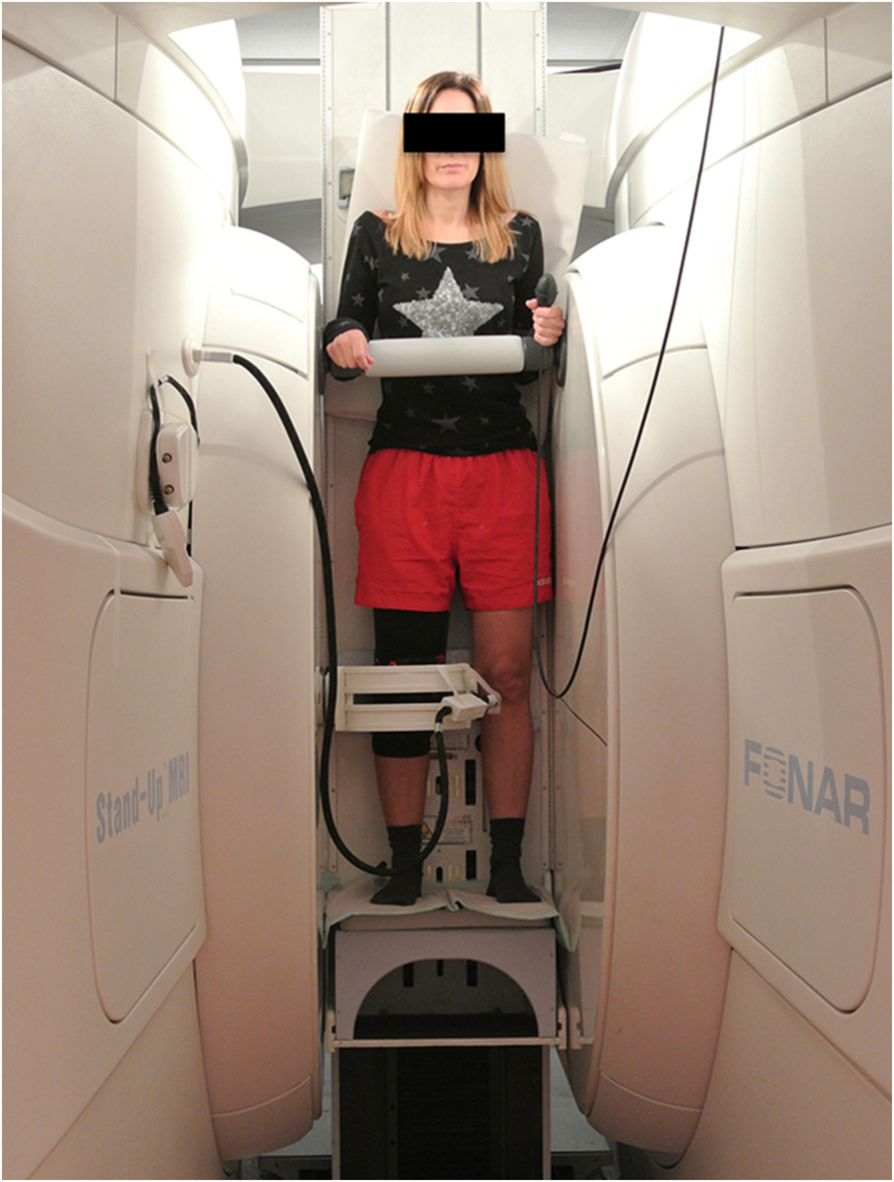
The upright open-MRI scanner with a subject examined at full extension weight-bearing condition

MRI assessment was performed using an open-configuration, 0.6 Tesla Upright MRI scanner (FONAR Inc., Melville, NY, USA) (Fig. 2). The subjects were examined in the upright weight-bearing position at full extension (0° flexion) and 15° and 30° flexion with and without the realignment brace at the same flexion angles. The order of the two trials was randomized. At all examined angles, the subjects were requested to keep the quadriceps engaged with both legs evenly loaded during scanning with assistance of a standard backrest and a bar in front of the chest. The flexion angles during the examinations were adjusted by using a standard goniometer and confirmed throughout the examination by using an MRI-compatible electronic goniometer (fMRI Compatible S700 1DOF Shape Sensor, Measurand Inc., Canada).

The following sequences were obtained: (1) 3-dimensional (3D) weighted gradient-echo sagittal images with an 8 × 29 × 9.6 cm field of view (FOV); slice thickness 1.5 mm; matrix 64 × 160 pixel with a pixel resolution of 1.25 × 1.25; echo time (TE) 5.6 ms, repetition time (TR) 11.2 ms, flip angle 40°; scan time ~50 s. (2) T1-weighted axial images with an 26 × 26 × 6.5 cm FOV; slice thickness 2.5 mm; matrix 416 × 416 pixel with a pixel resolution of 0.625 × 0.625; TE 15 ms, TR 140 ms, flip angle 90°; scan time ~135 s.

### Measurement of disease determinants

**Fig. 3:**
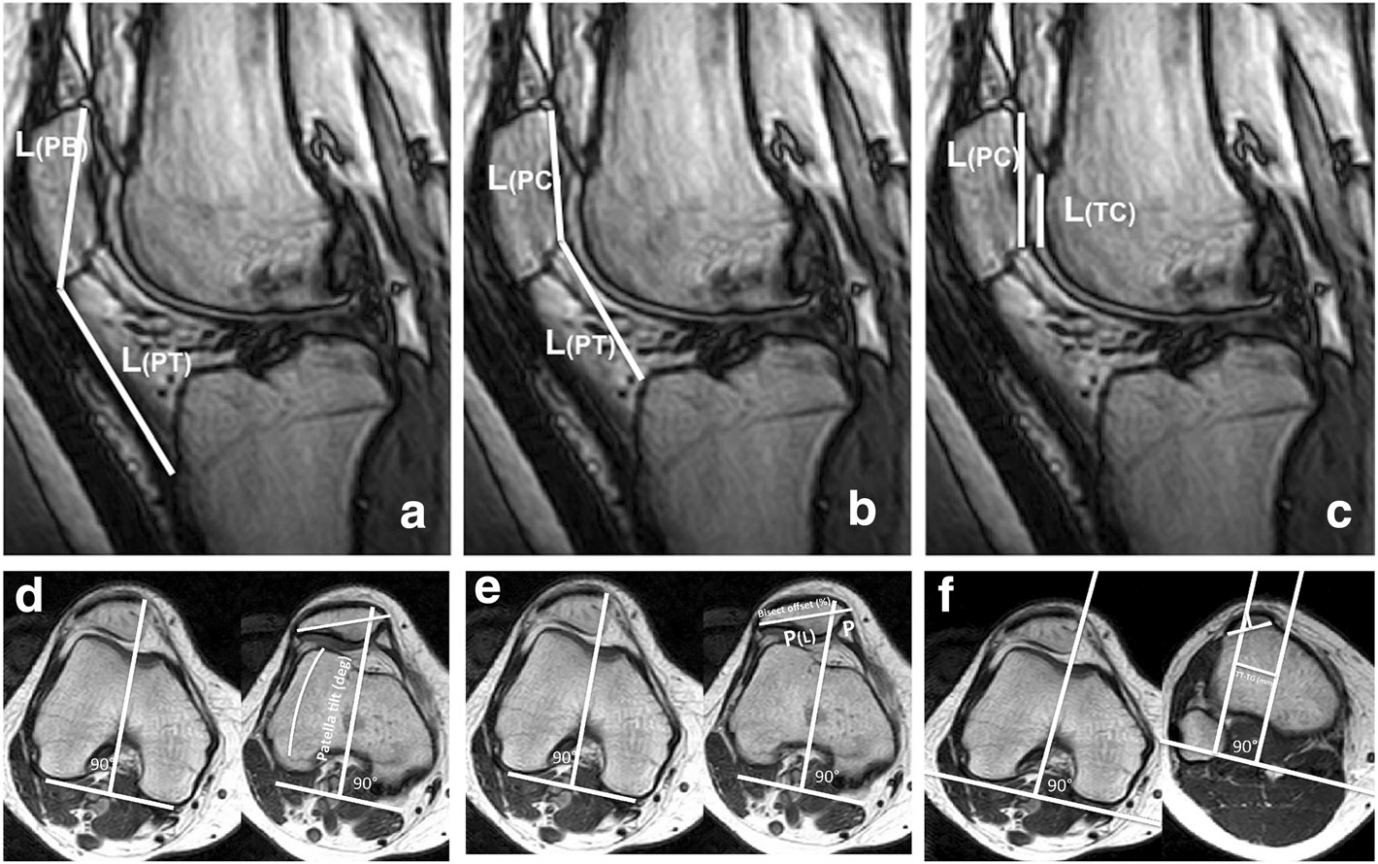
Descriptions of the measurement of the patellofemoral indices. **a** Insall–Salvati Index, L(PT) / L(PB). **b** Caton–Deschamps Index, L(PT) / L (PC). **c** Patellotrochlear Index (%), (L(TC) / L(PC)) × 100. **d** Patella tilt angle (°), the angle formed by lines joining the posterior femoral condyles and the maximum width of the patella. **e** Bisect offset (%), (P(L)/P) × 100. **f** TT–TG distance, distance between the midpoint of the patellar tendon at the insertion of the tibial tuberosity and a reference line through the first craniocaudal transverse slice that depicts complete cartilaginous trochlea coverage

Six commonly used disease determinants defined by patellofemoral indices (Insall–Salvati Index, Caton–Deschamps Index, Patellotrochlear Index, bisect offset, patella tilt angle, tuberositas tibiae–trochlear groove (TT–TG) distance) were assessed by MRI. These indices had been used in an earlier study evaluating the effects of weight bearing and knee flexion angle in subjects with patellofemoral instability [23]. To determine patella alta, patellar height was defined on the sagittal MR images by the Insall–Salvati Index (ISI), the Caton–Deschamps Index (CDI), and the Patellotrochlear Index (PTI) [24–26]. Patella alta is assumed to result in patellar maltracking and instability due to reduced medial–lateral constraint of the patella to the femoral trochlear groove, particularly at low knee flexion angles [8]. The ISI and CDI provide a measure of the height of the patella relative to the proximal tibia. The ISI determines patellar tendon length relative to the patella bone diagonal length (Fig. 3a). The CDI determines the distance from the anterosuperior border of the tibial plateau to the distal end of the patellar cartilage relative to the length of the patellar articular cartilage (Fig. 3b). Larger values indicate a higher position of the patella relative to the tibia. The PTI was determined to acquire a direct measure of patellar height relative to the femoral trochlea by calculating the ratio of the length of the patellar cartilage to the femoral trochlear articular cartilage overlapping the patellar cartilage (Fig. 3c). The index is reported as a percentage with larger values indicating greater contact area between the patellar and trochlear cartilages.

Further important factors as determinants of patellofemoral alignment are dependent on the geometry of the trochlear groove and rotation of the femur relative to the tibia. The patella tilt angle (PTA) [6, 27] and the bisect offset (BO) [6, 28] are used to quantify patellar rotation and mediolateral displacement of the patella relative to the femur. The TT–TG distance [2] determines the alignment of the extensor mechanism measured as the distance between the trochlear groove and the tibial tuberosity. These parameters were determined on the axial MR images. The PTA is defined as the angle between the patella and the posterior femoral condyles (Fig. 3d). Larger values indicate increased external rotation of the patella relative to the femur. The BO is reported as the percentage of the patella positioned lateral to the midline of the femur (Fig. 3e). Larger values indicate a more lateralized position of the patella relative to the femur. The alignment of the extensor mechanism was determined with the TT–TG, measured as the distance between the midpoint of the patellar tendon insertion at the tibial tuberosity and the first craniocaudal transverse slice that depicts complete cartilaginous trochlea coverage (Fig. 3f). Larger values indicate increased malalignment with increased lateral force displacement of the patella.

Measurements of the patellofemoral indices were made using OsiriX DICOM viewer software (Version 5.5.2; OsiriX Foundation, Geneva, Switzerland). All measurements were performed by one experienced investigator. The methodology was validated previously with good to excellent inter-rater reliability for all parameters [23].

### Statistical analysis

Analyses were conducted using SPSS (version 21.0; SPSS Inc., Chicago, IL, USA). All outcome parameters were tested for normal distribution. Due to the approximately normal distribution, means and standard deviations (SDs) were calculated for continuous variables (i.e., all outcome parameters). The effect of the brace and differences between the three angles were tested in a two-way analysis of variance (ANOVA) with two repeated measures (angle as one within-subjects factor and brace as the second within-subjects factor). If Mauchly’s sphericity test was significant, the Greenhouse–Geisser correction was used. Post hoc tests were calculated (paired *t* tests) after alpha adjustment according to Bonferroni.

## Results

**Fig. 4:**
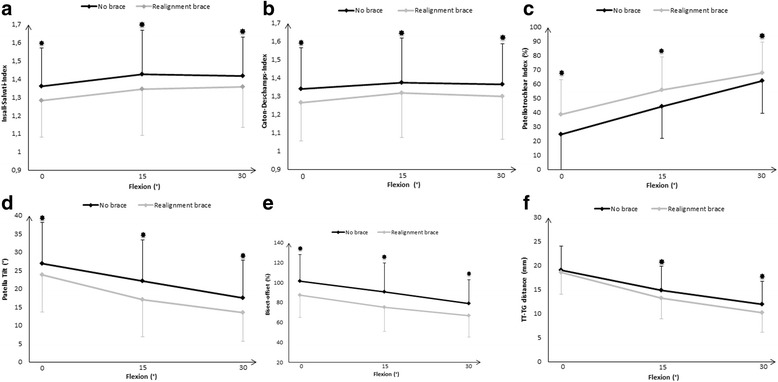
**a**–**f** Comparison of the patellofemoral indices with the realignment brace (*gray lines*) and without the brace (*black lines*) at full extension (0° flexion) and 15° and 30° flexion with upright weight-bearing condition. Data presented as mean ± SD. *Asterisks* indicate significant group differences based on post hoc pairwise comparisons (*p* ≤ 0.05); according to Bonferroni correction (0.05 / 36), a *p* < 0.0014 was considered as significant

A significant effect of the brace with reduction of all three patellar height ratios (all *p* ≤ 0.001) was observed. A significant brace-by-angle interaction (*p* = 0.012) was only found for the Patellotrochlear Index. Post hoc pairwise comparisons revealed reduced patellar height ratios at full extension and 15° and 30° flexion (all *p* ≤ 0.001) when using the realignment brace compared to the condition without the brace (Figs. 4a, 4b, and 4c).

A significant effect of the brace with reduction of the patella tilt angle and bisect offset (both *p* ≤ 0.001) was found. No significant brace-by-angle interaction was seen for either index. Post hoc pairwise comparisons revealed that the brace significantly reduced the patella tilt angle by 3.2° ± 2.8° at full extension, 5.0° ± 3.6° at 15° flexion, and 4.0° ± 4.3° at 30° flexion (all *p* ≤ 0.001) (Fig. 4d). The bisect offset was reduced by 14.2 ± 10.9 % at full extension, 15.6 ± 12.6 % at 15° flexion, and 12.3 ± 11.8 % at 30° flexion (all *p* ≤ 0.001), respectively (Fig. 4e). A significant effect of the brace with reduction of the TT–TG distance (*p* = 0.001) with a significant brace-by-angle interaction (*p* = 0.003) was observed. Post hoc pairwise comparisons revealed no significant differences of the TT–TG distances at full extension, but significant reductions of 1.7 mm ± 1.7 mm at 15° flexion (*p* = 0.001), and 1.7 mm ± 1.6 mm at 30° flexion (*p* ≤ 0.001) with the realignment brace compared to the condition without the brace (Fig. 4f).

**Fig. 5:**
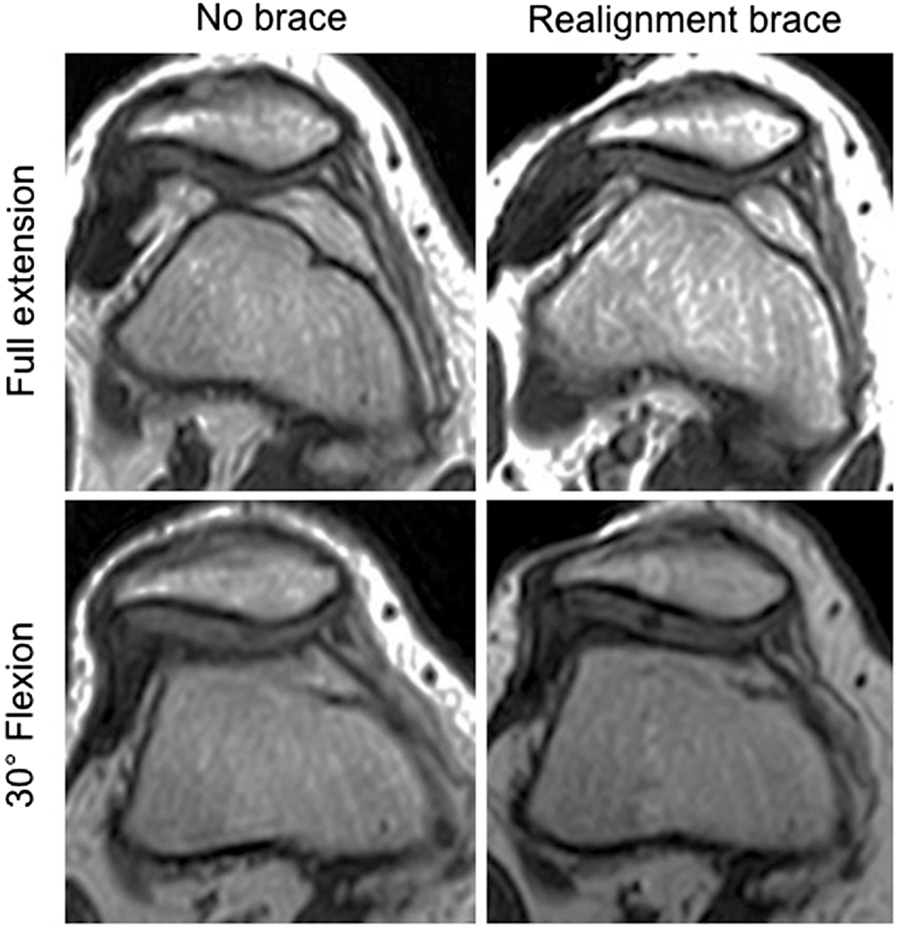
Axial imaging of a subject with patellofemoral instability with and without the realignment brace at 0° and 30°. The joint congruence appears considerably improved at the condition with the brace

## Discussion

The most important findings of the study are that the dynamic patellar realignment brace, Patella Pro, appears to be able of improving typical disease determinants used in MR imaging for lateral patellar instability at flexion angles between 0° and 30° in the upright weight-bearing condition. The strength of this study is that it found objective MRI data demonstrating the effect of the brace in patients with clinically significant patellar instability in the weight-bearing condition. The examined knee positions of full extension and 15° and 30° flexion reflect the clinically most important range of motion when the patella is not constrained by the trochlea. Since patellar motion is particularly influenced by quadriceps activation at extension and early flexion [29], it appears important that the evaluation of patellofemoral indices that have an influence on the treatment decisions is done during conditions with muscle activation such as weight bearing. Upright weight-bearing MRI has been shown to be a feasible method at different flexion angles [14, 23, 29] with good to excellent inter-rater agreement for the measurements of the parameters used in this study [23, 30, 31].

Among the conservative treatment options for patellofemoral pain and instability, taping and bracing of the patella to modify the patella position in order to improve patellar tracking have evolved as popular treatment modalities [14, 18, 32]. Furthermore, neuromotor and proprioceptive function can be improved [19, 33]. A meta-analysis published in 2002 revealed that the use of a patellar brace in patients with patellofemoral pain syndrome has positive effects on pain, function, and patellofemoral congruence angle compared with an untreated control group [32]. However, in another meta-analysis of 2008 to evaluate the evidence for patellar taping and bracing in the management of chronic knee pain, only limited evidence was reported to demonstrate the efficacy of patellar bracing [18]. In subjects with lateral patellar instability, the effects of stabilizing braces that exert a medially directed force that may counteract lateral patellar maltracking might be more pronounced. However, evidence to demonstrate the efficacy of patellar bracing for patellofemoral instability is limited.

The Patella Pro dynamic realignment brace used in this study was evaluated for biomechanical efficacy in an experimental setup using six fresh frozen human cadaver specimens tested in a knee simulator. Although not reaching statistically significance, average medialization of the patella of 1.04–1.66 mm in the tests with the brace compared to no brace was observed in 0°–45° flexion [21]. In the present study, it was demonstrated in vivo that the realignment brace significantly medialized the patella (reduction of BO) relative to the femur by 12.3–15.6 % and reduced the PTA by 3.2°–5.0° between 0° and 30° flexion. This is in line with the findings of a study evaluating the effects of a patellar realignment brace that consisted of a viscoelastic silicone insert with an integrated control guide designed to counteract patellar subluxation or dislocation during joint motion. In 19 patients with subluxation of the patella during active movement, loaded kinematic MR imaging, the authors reported that in 76 % of the included subjects, a qualitative correction of or improvement in patellar subluxation (i.e., centralization of the patella or a decrease in the displacement of the patella) occurred after application of the brace [17]. However, in a study evaluating 21 patients with clinical signs of patellar subluxation (*n* = 16) or dislocation (*n* = 5) using the same brace, no significant effect on the BO and PTA before or after wearing the patellar brace was found during active extension in the kinematic MR imaging [34]. Draper et al. evaluated the effects of a patellar-stabilizing brace in women with patellofemoral pain syndrome compared to a sleeve and a condition with no brace as in our study by real-time MRI in weight-bearing conditions in an open-bore MRI scanner [15]. The patellar-stabilizing brace significantly reduced the BO of pain subjects between 0° and 60° of knee flexion by an average of 4 % compared to the condition with no brace. The largest reduction of 6 % occurred at full extension. In the same study, the PTA of the pain subjects significantly decreased by 3° on average when using the brace at knee flexion angles between 0° and 20°. As for the BO, the largest reduction of the PTA (4°) occurred at full extension. Accordingly, in both parameters, the reduction was significantly better than with a sleeve [15]. In a comparable study, Powers et al. analyzed the BO and PTA comparing two different patellar braces and the no-brace condition using MRI in supine position with muscle activation forced by resistance put on the extensor mechanism using a custom-built loading apparatus that resembled a leg press machine [35]. As in the study of Draper et al., only females with patellofemoral pain syndrome were evaluated. On average, the amount of decrease in the BO was 3.6 and 2.4 % of patellar width for the two patellar braces, respectively. The greatest decrease of 4.8 % occurred at 20°. The average PTA was insignificantly reduced by 0.7° and 1.4°, respectively [35]. In summary, the brace effect on the reduction of the BO and PTA were greater in the present study than observed by the aforementioned studies [15, 35]. Possible reasons may be that different braces were used and the patients included in the studies of Draper et al. and Powers et al. did not present patellofemoral instability. In subjects with patellofemoral pain syndrome and no history of patellar dislocation, the BO and PTA are usually expected to be less pronounced than in patients with significant lateral patellofemoral instability, which is highlighted by the greater absolute values of these indices found in the present study.

With respect to the patellar height ratios, a significant reduction was observed at all evaluated angles by using the realignment brace in the present study. Since increased patellar height is acknowledged as a contributing factor to patellar instability [8, 36], the brace effect may be important to prevent subluxation and dislocation. Other aforementioned studies that evaluated brace effects with MRI did not consider the changes of patellar height. However, in a study of McWalter et al. evaluating 19 subjects with lateral patellofemoral osteoarthritis, a patellar realignment brace caused a significant distal translation of 1.09 mm in the loaded condition with static knee positions in a supine position with 15 % body weight pushed against a foot plate compared to 0.67 mm in the unloaded condition throughout 0°–50° flexion during MR imaging [16]. A possible explanation for the distalizing brace effect could be that weight-bearing results in a significant increase in patellar height ratios in subjects with patellofemoral instability [23]. This effect is less pronounced while wearing the brace as indicated by McWalter et al. who studied the brace effect with and without loading [16]. The tracking system of the Patella Pro, which is mounted on a hinged sleeve and covers the patella from the inferior over the medial to the superior aspect, obviously not only applies a dynamic, medially directed force but also additionally an inferiorly directed one in cases of patella alta when the patella is not in its designated space.

Accordingly, the TT–TG has not been considered in other studies evaluating brace effects. In the present study, the TT–TG was significantly reduced by the realignment brace at 15° and 30° flexion but not at full extension. The TT–TG is influenced by various factors, especially femoral and tibial rotation and trochlea dysplasia and, when increased, regarded as one of the important factors in patella instability [7, 31, 37, 38]. Since the patellar realignment brace does not directly influence any of these factors, the brace effect on the TT–TG appears surprising at first sight. However, with the reverse “screw-home” mechanism during early flexion of the knee, which leads to a medialization of the tibial tubercle and femoral external rotation [39, 40], the TT–TG decreases during flexion [23, 41, 42]. It was shown in an experimental study with knee cadaver specimens that activation of the vastus medialis leads to increased tibial internal rotation and thus a reduction of the TT–TG distance compared to no muscle activity [43]. Gilleard et al. described an earlier onset of the vastus medialis obliquus (VMO) activity when the patella was taped compared with the untaped condition during step-up and step-down tasks [44]. Thus, an explanation for the effect of the realignment brace could be a possibly improved VMO activity. Furthermore, since the patella is medialized by the brace with consecutive reduction of the Q-angle, tibial internal rotation or femoral external rotation might be more pronounced during flexion with a resulting decrease in TT–TG distance. However, as for the reduction of patellar height, more research is necessary to confirm these findings.

This study has the following limitations: (1) weight-bearing kinematics were evaluated in static upright standing conditions, which do not fully reflect the dynamic flexion kinematics of the lower limb. (2) Blinding of the investigator to the two conditions of brace or no-brace was not possible, since a certain degree of artifacts occurred in the area of the metal spring of the tracking system, which was outside of the area of interest for the assessment of the parameters. (3) Adding a further study condition with a simple sleeve without specific biomechanical patellar tracking function as a control for the dynamic patellar realignment brace could have reduced bias of wearing a brace and improved the assessment of the hypothesized biomechanical benefits of the patellar realignment brace. (4) The heterogeneity of the patterns that lead to lateral patellar instability in the included patients can be estimated by the standard deviations observed. Further evaluation may warrant clarification in which specific pathological patterns an effect of the brace may or may not be expected, respectively.

## Conclusion

From this study, it can be concluded that the dynamic patellar realignment brace, Patella Pro, may be able to improve disease determinants in patients with lateral patellofemoral instability in the upright weight-bearing condition at 0°–30° flexion. If clinical symptoms can be meaningfully reduced and subluxation or dislocation can be prevented warrants further investigation.
